# Experimental investigation of non-Hermitian physics in the quantum regime

**DOI:** 10.1093/nsr/nwaf144

**Published:** 2025-04-18

**Authors:** Yang Wu, Yunhan Wang, Xing Rong, Jiangfeng Du

**Affiliations:** Institute of Quantum Sensing and School of Physics, Zhejiang University, China; Zhejiang Key Laboratory of R&D and Application of Cutting-edge Scientific Instruments, Zhejiang University, China; Institute of Fundamental and Transdisciplinary Research, Zhejiang University, China; CAS Key Laboratory of Microscale Magnetic Resonance and School of Physical Sciences, University of Science and Technology of China, China; Hefei National Laboratory, University of Science and Technology of China, China; CAS Key Laboratory of Microscale Magnetic Resonance and School of Physical Sciences, University of Science and Technology of China, China; Zhejiang Key Laboratory of R&D and Application of Cutting-edge Scientific Instruments, Zhejiang University, China; Hefei National Laboratory, University of Science and Technology of China, China; Institute of Quantum Sensing and School of Physics, Zhejiang University, China; CAS Key Laboratory of Microscale Magnetic Resonance and School of Physical Sciences, University of Science and Technology of China, China; Zhejiang Key Laboratory of R&D and Application of Cutting-edge Scientific Instruments, Zhejiang University, China; Institute of Fundamental and Transdisciplinary Research, Zhejiang University, China; Hefei National Laboratory, University of Science and Technology of China, China

## Abstract

This perspective presents advances in non-Hermitian physics within quantum systems, covering experimental realizations across platforms and applications, along with proposed future research directions.

The concept of non-Hermiticity and related counterintuitive aspects have raised intense attention in the past two decades and have been extensively explored in the interdisciplinary field of non-Hermitian physics combined with different kinds of non-conservative systems not limited to the optical and acoustic arrangements where the wave dynamics dominates, but even the electronic, mechanical systems and the diffusive system of heat. What makes this class of physical systems so attractive is the parity-time (PT) symmetry breaking and the notions of singularities known as exceptional points (EPs). These unique features of non-Hermitian systems, which have no Hermitian counterpart, lead to a plethora of intriguing behaviors due to the dramatically altered overall response near EPs and exotic functionalities based on the great ability of state control (see Fig. 1 for exemplary quantum systems and applications).

**Figure 1.**
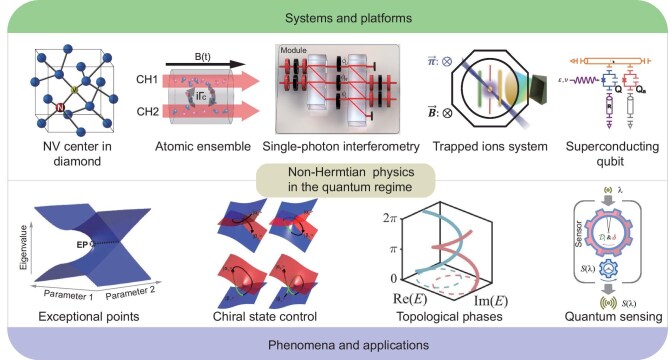
Investigations of non-Hermitian physics in various quantum systems and the exotic phenomena and potential applications of non-Hermitian physics in the quantum regime. The figures are adopted from [1–5,8–10,15] with permissions.

In non-Hermitian systems, the simultaneous coalescence of *k* ($k \ge 2$) eigenvalues and their corresponding eigenstates defines a class of singularities known as *k*th-order exceptional points (EP*k*s). EPs exhibit unique physical phenomena and applications [1], such as unidirectional invisibility, single-mode lasing and sensitivity enhancement. These singularities serve as fundamental constructs for comprehending topological phase transitions and enabling precision manipulation of quantum states. Significant experimental progress has been made in investigating EPs within a variety of quantum systems. For example, observation of an EP has been implemented in the nitrogen-vacancy (NV) center single-spin system [2], which is achieved by constructing a non-Hermitian Hamiltonian through the dilation method. In addition, in trapped ions [3], superconducting qubits [4] and atomic ensemble systems [5], a non-Hermitian Hamiltonian can be constructed by introducing dissipation to achieve observation of EPs. Furthermore, based on the photonic system, observation of the EP is achieved by the evolution process under the non-Hermitian Hamiltonian, which is realized by constructing a non-unitary operation [6].

Recently, high-order EPs have been proposed to host features beyond EP2s. High-order EPs are beneficial to improve the sensing performance of a non-Hermitian system. In addition, non-trivial topological features are expected to be more abundant as the order of the EPs increases. These advantages have triggered the pursuit of constructing high-order EPs. EP3s have been observed in quantum optical systems simulating a two-dimensional non-Hermitian system in the reciprocal space with single photons [7]. These approaches can be applied to explore topological physics related to high-order EPs and offer new potentials for applications in quantum technologies.

An interesting dynamical quantum effect in the non-Hermitian quantum system is the chiral state transfer related to EPs, where the final state after the evolution of encircling an EP cannot recover the original state in one direction with a single loop, in stark contrast to the adiabatic evolution of accidental degeneracies in Hermitian systems. This phenomenon is linked to the non-trivial topological features of EPs that have been demonstrated in the NV center of diamond [8]. In addition, Kibble–Zurek theory is experimentally verified by dynamically evolving across the EP in a single-photon interferometry setup [9]. Both the defect density and its fluctuations are confirmed to follow universal scaling, which allows us to extract the correlation length and dynamical exponents of the underlying EP.

Novel topological phenomena of non-Hermitian systems are also studied. For example, non-Hermitian topological structures manifested by complex eigenvalues have been observed, which are intrinsic to non-Hermitian systems [10]. The non-Hermitian bulk-boundary correspondence beyond the conventional framework is demonstrated in the discrete-time non-unitary quantum-walk dynamics of single photons [11]. The intriguing localization of nominal bulk states at boundaries, known as the non-Hermitian skin effect, suggests a non-Bloch band theory in non-Hermitian systems. Very recently, the investigation of the topological physics of non-Hermitian systems has been demonstrated in the high-dimensional system [12]. The two-dimensional skin effect is observed by creating a two-dimensional non-Hermitian topological band for ultracold fermions in spin-orbit-coupled optical lattices.

Based on the unique spectral structure of the non-Hermitian system, intense research effort has focused on exploring the possibilities of enhancing the sensitivity of quantum sensing. A quantum realm of the EP-enhanced sensor with heterodyne detection has been achieved in a setup consisting of coupled active and passive optical modes [13]. EP-enhanced quantum sensing has also been demonstrated in the loss-enhanced magneto-optical effect [14], where the frequency splitting exhibits enhancement compared to the Hermitian counterpart. The spatial motion of spins in the PT-broken phase could be a resource for improving measurement stability. Recently, universal non-Hermitian sensing has been experimentally demonstrated in the absence of EPs [15]. The sensitivity of the probe shows superior performance under background noises that cannot be suppressed through repetitive measurements.

The simplest implication of non-Hermitian physics in the quantum regime is observing the non-Hermitian phenomenon that occurs in a quantum system, which has been extensively studied in theory and experimentally demonstrated in diverse quantum platforms, as discussed above. Nevertheless, this interpretation cannot offer more physical insights unless we inquire about the different features in quantum systems from that in classical systems. Therefore, one future direction involves studying non-Hermitian physics in the presence of quantum effects, such as quantum jumps and dephasing. This will inspire research into the integration of non-Hermitian physics with non-equilibrium physics, potentially leading to cutting-edge applications like time crystals and frequency combs. Furthermore, the introduction of non-Hermiticity also alters the properties of quantum systems, holding promise for applications in quantum control and quantum sensing. The unique dynamical behaviors in non-Hermitian systems demonstrate robustness against noise, which could facilitate precise quantum control, particularly in open quantum systems where challenges persist. The enhanced response of non-Hermitian systems to weak signals requires further experimental verification and the development of practical quantum devices.
